# Histopathological Findings in COVID-19 Cases: A Systematic Review

**DOI:** 10.7759/cureus.25573

**Published:** 2022-06-01

**Authors:** Hamed Hammoud, Ahmed Bendari, Tasneem Bendari, Iheb Bougmiza

**Affiliations:** 1 Preventive Medicine, Hamad Medical Corporation, Doha, QAT; 2 Department of Pathology, Lenox Hill Hospital, New York, USA; 3 Faculty of Medicine, Zagazig University, Zagazig, EGY; 4 Community Medicine Residency Program, Primary Health Care Corporation, Doha, QAT

**Keywords:** covid-19, forensic pathology, autopsy, histopathology, sars-cov-2

## Abstract

The ongoing coronavirus disease 2019 (COVID-19) pandemic has turned into one of the most serious public health crises of the last few decades. Although the disease can result in diverse and multiorgan pathologies, very few studies have addressed the postmortem pathological findings of COVID-19 cases. Active autopsy findings amid this pandemic could be an essential tool for diagnosis, surveillance, and research. We aimed to provide a comprehensive picture of the severe acute respiratory syndrome coronavirus 2 (SARS-CoV-2) histopathological features of different body organs through a systematic review of the published literature. A systematic search of electronic databases (PubMed, ScienceDirect, Google Scholar, medRxiv, and bioRxiv) for journal articles of different study designs reporting postmortem pathological findings in COVID-19 cases was performed. The Preferred Reporting Items for Systematic Reviews and Meta-Analyses (PRISMA) guidelines were used for conducting the review. A total of 50 articles reporting 430 cases were included in our analysis. Postmortem pathological findings were reported for different body organs: pulmonary system (42 articles), cardiovascular system (23 articles), hepatobiliary system (22 articles), kidney (16 articles), spleen and lymph nodes (12 articles), and central nervous system (seven articles). In lung samples, diffuse alveolar damage (DAD) was the most commonly reported finding in 239 cases (84.4%). Myocardial hypertrophy (87 cases, 51.2%), arteriosclerosis (121 cases, 62%), and steatosis (118 cases, 59.3%) were the most commonly reported pathological findings in the heart, kidney, and the hepatobiliary system respectively. Autopsy examination as an investigation tool could lead to a better understanding of SARS-CoV-2 pathophysiology, diagnosis, and management, subsequently improving patient care.

## Introduction and background

The novel coronavirus disease 2019 (COVID-19) pandemic has become one of the most challenging public health crises for decades. It first emerged in Wuhan, China, in late December 2019 and is believed to be caused by infection with the severe acute respiratory syndrome coronavirus 2 (SARS‐CoV‐2) virus [1]. The first cases of COVID-19 in China were believed to be of zoonotic origin, but the global spread of the disease has been mainly travel-related. The disease has spread from China to nearly 200 countries worldwide [2]. The virus is easily transmissible via droplets and fomites or when bodily fluids of the infected individual come into contact with another person’s face, mouth, eyes, or nose [3].

Regarding the pathogenesis of COVID-19, angiotensin-converting enzyme 2 (ACE2), which is highly expressed in the respiratory tract, acts as a receptor to SARS-CoV-2. The virus invades the human cells, causing massive destruction and inflammation of different organs and subsequently affecting the vascular supply and even progressing to fibrosis [4]. The main clinical manifestations include fever, cough, fatigue, and shortness of breath. Other less common symptoms include headache, sore throat, and rhinorrhea. Also, one-fifth of patients (20%) presented with severe symptoms such as respiratory failure, multiorgan failure, and septic shock, all of which necessitate intensive care [5]. Although COVID-19 mainly affects the respiratory system, there have been reported cases of cardiogenic and renal involvement in patients without previously known heart or renal diseases [6,7].

The case-fatality rate for COVID-19 is variable across different nations and ranges from 11.75% in Italy to 0.37% in South Africa. The mean recovery time is two weeks for mild cases and three to six weeks for severe or critical cases [8]. The diagnosis of COVID-19 relies mainly on reverse-transcription polymerase chain reaction (RT-PCR) with some emerging evidence endorsing the utility of characteristic CT and laboratory findings [9]. COVID-19 is a member of the coronavirus family, which includes Middle East Respiratory Syndrome-related coronavirus (MERS-CoV) and SARS-CoV [10]. Both MERS-CoV and SARS-CoV are believed to affect humans and cause interstitial pneumonia, pneumocyte hyperplasia, and acute diffuse alveolar damage (DAD) [11,12]. The diverse histopathological findings associated with COVID-19 infections suggest that multiple organs are affected by the virus, with the pulmonary system being the most common system to be affected. Carsana et al. showed in their study the wide variety of pathological findings related to COVID-19 in the respiratory system. They found that pneumocytes desquamation, pulmonary edema, and DAD are the most common microscopic findings [13].

As of August 10, 2020, the number of COVID-19 cases worldwide has surpassed 20,162,474 million, with almost 737,417 deaths. However, the number of studies addressing the postmortem autopsy findings of COVID-19 patients is still scarce given the number of deaths. This could be attributed to the fears of contagion associated with COVID-19 infection. Since the beginning of the pandemic, the Centers for Disease Control and Prevention (CDC) have released interim guidelines for the collection and analysis of clinical specimens that might contain SARS-CoV-2 [14].

Active autopsy findings amid emerging epidemic diseases have been identified as an essential tool for diagnosis, surveillance, and research. Pathologists are usually among the first healthcare professionals to identify novel infectious agent outbreaks [15]. Our aim in this systematic review is to provide a comprehensive picture of the SARS-CoV-2 histopathological features of different body organs in postmortem autopsies through a systematic search of the published literature. We believe that this will foster a better understanding of the mechanisms of injury and pathophysiology of severe SARS-CoV-2 infection and subsequently improve patient care.

## Review

Methods

This study followed the recommendations established by the Preferred Reporting Items for Systematic Reviews and Meta-Analyses (PRISMA) statement (Appendix 1) [16].

Sources of Information

A predetermined protocol was used to perform this systematic review by using the following databases: PubMed, Google Scholar, ScienceDirect, and medRxiv. The reference lists of relevant studies were manually searched to identify cited articles that were not captured by electronic search.

Selection Criteria

Articles were included if they met the following eligibility criteria: (1) addressed pathological reports of COVID-19 autopsies or postmortem cases, (2) involved human subjects (at least one case), (3) all study designs were involved (case report, case series, cross-sectional, case-control, randomized and non-randomized studies), and (4) no language restrictions were applied.

Study Selection and Search Terms

The search terms and keywords across the different databases have been provided in Appendix 2. The selection was broad enough to include as many studies as possible. In the initial phase, two independent reviewers (H.H. and A.B.) screened the titles and abstracts of the articles by using the Rayyan QCRI® website [17]. As a result, all non-relevant articles were excluded. In the second phase, the full texts of the remaining articles were independently reviewed for the final selection of eligible studies. Any disagreement between the two reviewers was resolved by a third reviewer (T.B.).

Quality Assessment and Risk of Bias

To assess the internal validity of the included studies, we used different tools according to the study design. For cross-sectional studies, the Newcastle-Ottawa Quality Assessment Scale (NOS) (modified for cross-sectional studies) was used after removing items that relate to comparability and adjustment. The tool contains three major subsections (Selection, Comparability, and Outcome). A score for quality, modified from the tool, was used to assess the appropriateness of study design, recruitment strategy, sample representativeness, reliability of the outcome, sample size provided, and appropriate statistical analyses. At least two reviewers (H.H., A.B., T.B.) independently ranked these domains. When the independent evaluations of the ranks differed between the two reviewers, they discussed disagreements to reach a consensus. For case reports and case series, a version of the NOS checklist has been adapted by Murad et al. to assess the methodological quality of case reports and case series [18]. By this approach, we assessed the quality of each study with regard to four domains: selection, ascertainment, causality, and reporting. From the results of each checklist, if 25% or less of the criteria were addressed, the article was scored as poor; if 26-50% of the criteria were addressed, the article was scored as fair; if 51-75% of criteria were addressed, the article was scored as good; and if 76-100% of the criteria were addressed, the article was scored as excellent.

Data Extraction

A single author (A.B.) extracted the variables from each included study. The data from the final list of included articles were transferred onto an online Google Sheet. Several study characteristics were extracted, including:

General characteristics such as study type, country of origin, article language, and sample size.

Study population demographics like age and gender.

Clinical findings like symptomatology, lab findings, and patient comorbidities.

Histopathologic and microscopic findings of different organs.

Results

**Figure 1:**
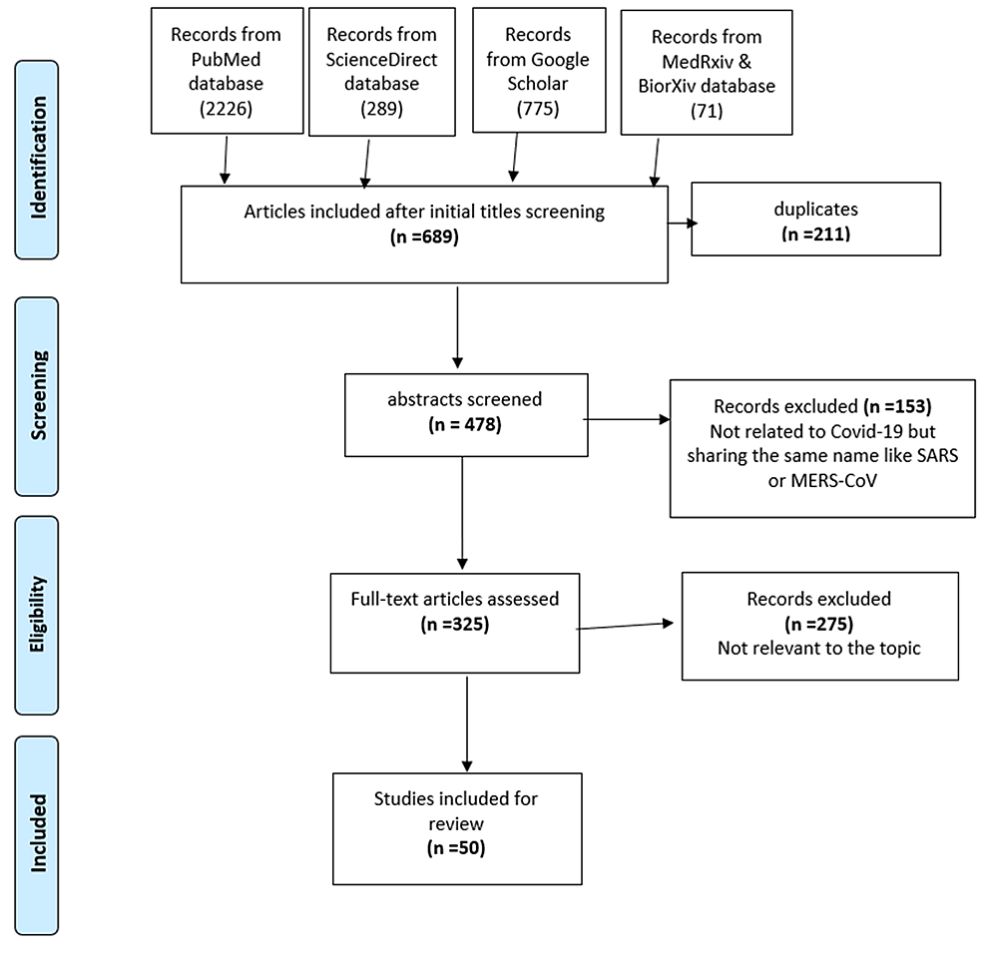
Flow chart showing the procedure for the selection of studies

Figure 1 shows a flow chart illustrating the procedure for the selection of studies. Initially, we identified 3,297 studies from five databases as follows; PubMed (2,262), ScienceDirect (189), medRxiv and bioRxiv (71), and Google Scholar (775). After the initial title screening, we were left with 689 articles; 211 duplicates were removed, leaving 478 for abstract screening. After excluding non-relevant articles, 50 articles were available for the final analysis.

Characteristics of Included Studies

**Figure 2:**
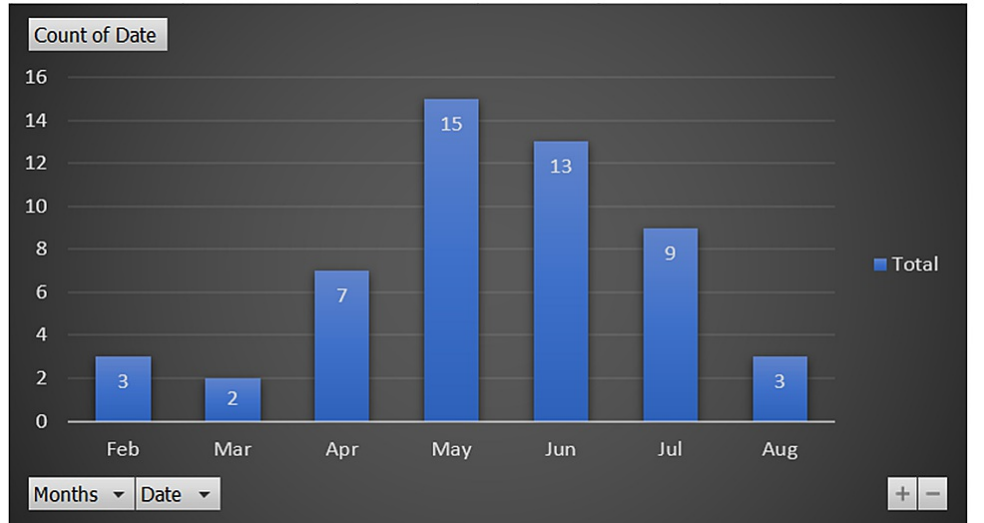
Timeline distribution of published articles

A total number of 50 studies were included in our systematic review, with 430 cases overall. Regarding the date of publication, only three studies were published in February, two in March, and seven in April, while 15 were published in May, which is the month with the highest number of publications (Figure 2). Regarding the type of published articles, case reports and case series were the most common categories (39 studies), followed by cross-sectional studies (10 studies), and there was one cohort study. Regarding the country of origin of the published studies, there were about 16 countries. The USA was the country with the most publications (16 studies), followed by China (10 studies), Germany (six studies), Italy (five studies), Switzerland (two studies), and there was one study from each of the following countries: Iran, Finland, Austria, Belgium, Japan, Spain, Netherlands, UK, Romania, Austria, and Denmark. As for the language of the published articles, only English and Chinese were represented: 47 studies were in English while three were in Chinese (Table 1).

**Table 1:** General characteristics of the included studies

Authors	Country	Language	Design	Number of cases	Study quality
Yao et al. [49]	China	Chinese	Case report	3	Excellent
Barton et al. [24]	USA	English	Case report	2	Good
Xu et al. [25]	China	English	Case report	1	Good
Tian et al. [27]	China	English	Case series	4	Good
Tian et al. [26]	China	English	Case report	2	Good
Su et al. [28]	China	English	Cross-sectional study	26	Good
Carsana et al. [13]	Italy	English	Cross-sectional study	38	Good
Schaller et al. [20]	Germany	English	Cross-sectional study	10	Fair
Menter et al. [50]	Switzerland	English	Cross-sectional study	21	Excellent
Edler et al. [51]	Germany	English	Cross-sectional study	12	Excellent
Remmelink et al. [52]	Belgium	English	Cross-sectional study	17	Excellent
Buja et al. [29]	USA	English	Case series	3	Good
Bradley et al. [30]	USA	English	Case series	14	Good
Lax et al. [53]	Austria	English	Cross-sectional study	11	Excellent
Wichmann et al. [31]	Germany	English	Cohort study	12	Good
Rapkiewicz et al. [21]	USA	English	Case series	7	Fair
Martines et al. [32]	USA	English	Case series	8	Good
Magro et al. [54]	USA	English	Case series	5	Excellent
Fox et al. [33]	USA	English	Case series	10	Good
Bryce et al. [55]	USA	English	Case series	25	Excellent
Prilutskiy et al. [34]	USA	English	Case series	4	Good
Konopka et al. [22]	USA	English	Case report	1	Fair
Fitzek et al. [56]	Germany	English	Case report	1	Excellent
Zhang et al. [35]	China	English	Case report	1	Good
Li et al. [36]	USA	English	Case report	1	Good
Cipolloni et al. [37]	Italy	English	Case report	2	Good
Adachi et al. [57]	Japan	English	Case report	1	Excellent
Flikweert et al. [58]	Netherlands	English	Case series	7	Excellent
Grillo et al. [38]	Italy	English	Case series	8	Good
Xu et al. [39]	China	Chinese	Case series	10	Good
Wu et al. [40]	China	Chinese	Case series	10	Good
Youd et al. [41]	UK	English	Case series	9	Good
Konopka et al. [23]	USA	English	Case series	8	Fair
Schaefer et al. [60]	USA	English	Case series	7	Excellent
Ackermann et al. [59]	USA	English	Case series	7	Excellent
Bösmüller et al. [61]	Germany	English	Case series	4	Excellent
Suess et al. [42]	Switzerland	English	Case report	1	Good
Sonzogni et al. [62]	Italy	English	Cross-sectional study	48	Excellent
Wang et al. [63]	China	English	Cross-sectional study	2	Excellent
Bruni et al. [43]	Italy	English	Case report	1	Good
Colmenero et al. [44]	Spain	English	Case series	7	Good
Beigmohammadi et al. [45]	Iran	English	Case series	7	Good
Heinrich et al. [46]	Germany	English	Case report	1	Good
Wang et al. [47]	China	English	Case report	2	Good
Ducloyer et al. [64]	France	English	Case report	1	Excellent
Kantonen et al. [65]	Finland	English	Case series	3	Excellent
Reichard et al. [48]	USA	English	Case report	1	Good
Cîrstea et al. [66]	Romania	English	Case report	1	Excellent
Schwensen et al. [67]	Denmark	English	Case report	1	Excellent
Santoriello et al. [68]	USA	English	Cross-sectional study	42	Excellent

Quality of Evidence

We used the GRADE framework for judging the precision and confidence estimate in the review. Generally speaking, the evidence derived from observational studies was classified as low quality [19]. Regarding the risk of bias assessment in the review, four articles scored between 26 and 50%, which is considered “Fair” [20-23]; 26 articles scored between 51 and 75%, which is considered “Good” [13,24-48], and 20 articles scored more than 76%, which is considered “Excellent” [49-68]. A high degree of inconsistency was noticed in the review as the study populations were heterogeneous with respect to main characteristics like age, gender, and comorbidities. Although there were no language restrictions applied in the review, publication bias may appear due to the fact that the number of published articles was small, especially at the beginning of the pandemic. Moreover, a very small number of countries were reporting autopsy findings. Regarding the indirectness, the majority of included studies used the same tool in diagnosing COVID-19, which is RT-PCR, the same tool in identifying histopathological findings, and the studied population varied between studies. Hence, the quality of evidence was rated as “Moderate” (Appendix 4).

Clinical Findings of the Cases

The review described a total of 430 patients with COVID-19. Among the included patients, gender was reported in 349 [297 males (85.1%) and 133 (14.9%) females]. The ages of the patients for whom age was reported ranged from 11 to 94 years. Regarding the presenting symptoms of patients whose clinical symptoms were reported (192 patients), fever was reported in 121 patients, followed by cough in 103 patients, and dyspnea in 91 patients [20-27,29-33,35,37,39-43,45-49,52-54,56-58,60,61,63-67]. Regarding the pre-existing comorbidities in patients whose medical history was reported, hypertension was found in 210 patients (48.8%), followed by coronary heart disease in 190 (44%), diabetes in 134 patients (31%), chronic kidney disease in 96 patients (22.3%), obesity in 64 patients (14.8%), chronic lung disease in 54 patients (12.5%), and cancer in 50 patients (11.6%) [13,20,21,23-33,35,39-42,45-66,68]. Regarding the organs included, this review observed the reported histopathology of different organs as follows: lungs and the pulmonary system were the most commonly described organs (42 articles) [13,20-27,29-33,35-38,40-42,45-47,49-61,63-67], followed by the heart in 23 articles [20,21,24,25,27,29,30,32,33,41,42,45-47,49-53,55,64,66,67], liver in 21 articles [20,21,24,25,27,29,30,32,42,45,46,49-53,55,62-64,66], kidneys in 16 articles [21,24,28-30,32,47,49-53,55,57,66,68], spleen and lymph nodes in 12 articles [21,29,30,32,34,39,50,51,53,55,57], central nervous system (CNS) in seven articles [20,30,46,48,52,55,65], skin in two articles [44,54], gall bladder in one article [43], and pharynx in one article [51].

Laboratory Investigations

In all of the included studies, RT-PCR on the nasopharyngeal swab was the predominant method used to confirm the positivity of COVID-19. RT-PCR on endotracheal aspirate swab was reported in one patient [67]. RT-PCR on skin biopsy was reported in seven patients [44]. Chest imaging, whether CT or X-ray, was reported in 206 patients (47.9%) [13,20,22,24-33,35,40,45,47,48,51,53,54,56-60,63,64,67]. Postmortem RT-PCR of the lung parenchyma was reported in 91 cases (21%) [23,24,30,32,35,37,40,41,46,47,51,52,58,64,66].

Lung Histopathological Findings

In the 42 articles that described lung pathology, the most commonly reported pathological findings were diffuse alveolar injury in 239 cases (84.4%) [13,20-27,29-33,35-38,40-42,45-47,49-52,55-61,63-67], followed by hyaline membrane formation in 184 cases (65%) [13,20-25,27,29,31,33,36,37,40-42,45-47,49-58,61,63,64,67], lymphocyte and/or monocyte infiltrates in 172 cases (60.7%) [13,20,21,24-27,29-31,33,35-37,40-42,45-47,49-56,58,61,64,66,67], pneumocyte hyperplasia in 171 cases (60.4%) [13,20,22,26,27,29,30,35,37,40-42,45-47,49,51-61,64,66], pulmonary microthrombi in 151 cases (53.3%) [13,21-23,29,31-33,36-38,46,49,51-53,55,56,58-61,65,66], fibrin exudation in 112 cases (39.8%) [13,21,22,29,33,35,37,40-42,45,47,49,51,52,54,56,58,61,63], lung fibrosis in 97 cases (34.2%) [13,20,26,30,31,33,35,38,49,51,52,56-58,63,67], intra-alveolar neutrophilic infiltration in 92 cases (32.5%) [13,20,22,24,27,29,31,33,37,40,42,47,49-51,54,58,61], intra-alveolar hemorrhage in 86 cases (30.4%) [13,27,29,30,32,33,42,45,47,49,50,52,54,57,66], interstitial thickening in 80 cases 28.3%) [13,20,26,27,30,32,35,37,41,49,56,59,61,66], vascular congestion in 77 cases (27.2%) [13,21,24,26,27,29,31,40,42,46,49,50,54,57], pneumocyte damage in 73 cases (25.8%) [13,21,25,27,33,35,47,49,59,61,63], squamous metaplasia in 68 cases (24%) [13,20,29,31,32,38,45,47,51,57,60,61,67], viral inclusion in 45 cases (15.9%) [13,26,30,32,49,55,61], serous exudation in 19 cases (6.7% [47,49,52,59], fibrinoid vascular necrosis in 17 cases (6%) [20,27,45,50,54,61], and pulmonary embolism in 14 cases (4.9%) [29,30,41,50,55,58] (Appendix 3).

Heart Histopathological Findings

In the 23 articles that described heart pathology, the most commonly reported pathology was myocardial hypertrophy in 87 cases (51.2%) [27,29,30,32,49,50,52,53,55], followed by myocardial fibrosis in 85 cases (50%) [27,29,30,32,33,41,46,47,51-53,55], coronary small vessel disease in 44 cases (25.9%) [21,24,29,41,50,53,55,64], myocardial cell infiltrate in 27 cases (15.9%) [20,21,25,45,49,51,53,55,66], cardiac amyloidosis in 10 cases (5.9%) [30,50,53], and myocardial necrosis in nine cases (5.3%) [45,47,49,50] (Appendix 3).

Liver Histopathological Findings

In the 21 articles that described liver pathology, the most frequently reported pathology was steatosis in 118 cases (59.3%) [21,24,25,27,29,30,32,34,42,45,46,50,52,53,55,62-64,66], followed by fibrosis in 62 cases (31.1%) [20,29,53,55,62,66], hepatic congestion in 59 cases (29.6%) [30,34,45,51-53,66], cellular infiltrate in 54 cases (27.1%) [20,25,27,30,53,62,63,66], hepatic necrosis in 44 cases (22.1%) [21,27,30,42,45,49,53,62,63], cholestasis in eight cases (4%) [53], and cirrhosis in four cases (2%) [27,52] (Appendix 3).

Kidney Histopathological Findings

In the 16 articles that described kidney pathology, the predominantly reported pathology was arteriosclerosis in 121 cases (62%) [24,28-30,51,55,68], followed by nephrosclerosis in 91 cases (46.7%) [24,30,55,68], acute tubular injury in 86 cases (44.1%) [21,28,32,49,50,53,55,66,68], glomerulosclerosis in 70 cases (35.9%) [28-30,32,47,52,53], tubular cast in 38 cases (19.5%) [21,28,30,49,52], glomerular fibrin thrombus in 21 cases (10.7%) [21,28-30,55,57,66,68], and viral particles in 16 cases (8.2%) [21,28,29] (Appendix 3).

Immune System (Spleen and Lymph Node) Histopathological Findings

In the 12 articles that described spleen pathology, the most commonly reported pathology was lymphocyte depletion in 38 cases (31.4%) [21,29,30,39,49,53,55], followed by hemophagocytosis in spleen in 12 cases (9.9%) [34,39,55,57]. In the 11 articles that described lymph node pathology, the most common pathology that had been reported was lymphocyte depletion in 23 cases (20.7%) [21,34,49,53,55], followed by hemophagocytosis in the lymph nodes in 22 cases (19.8%) [30,32,34,55,57].

CNS Histopathological Findings

In the seven articles that described CNS pathology, the most commonly reported pathology was cerebral hemorrhage in 11 cases (15.5%) [30,48,52,65], followed by focal spongiosis in 11 cases (15.5%) [48,52], vascular congestion in 11 cases (15.5%) [52,55], and diffuse or focal ischemic necrosis in nine cases (12.7%) [52,55].

Skin Histopathological Findings

In the two articles that described skin pathology, the most commonly reported pathology was thrombogenic vasculopathy in four cases (10, 0.4%) [44,54], followed by perivascular inflammation in two cases (10, 0.2%) [44,54], and vasculitis in one case [44].

Gall Bladder Histopathological Findings

In the one article that described gall bladder postmortem pathology, inflammatory infiltration and endoluminal obliteration of vessels with wall breakthrough, hemorrhagic infarction, and nerve hypertrophy were reported in a single case [43].

Pharynx Histopathological Findings

One study described pharyngeal postmortem pathology. The study included eight cases, seven of which reported mild to pronounced lymphocytic pharyngitis [51].

Discussion

This systematic review identified 50 studies with a total of 430 patients and postmortem pathological findings of different body organs. Since the beginning of the pandemic, the body of evidence and the number of published studies have increased over time, but it is still limited compared to the number of COVID‐19 deaths (almost one million deaths). There were only 16 countries that contributed to publishing autopsy reports of the COVID-19 deaths, which is considered very low given the fact that the disease affected nearly 200 countries all over the world [2]. With regard to the timeline of the published studies, it took us five months (up to May 2020) to find an appropriate number of publications addressing the postmortem pathological findings. One of the main reasons for the scarcity of published literature is the fear of COVID-19 infection transmission during postmortem examinations and the perceived risk among healthcare professionals, especially pathologists, about this "new" disease, coupled with a poor understanding of its pathological mechanism, especially at the beginning of the pandemic [69]. Moreover, in some countries, the number of safe autopsy rooms is very low, which, according to the WHO and CDC guidelines, is considered one of the barriers that contributed to the scarcity of evidence [70-72].

Postmortem Pulmonary Findings

Regarding the postmortem pulmonary pathology, our review showed that various histopathological findings had been identified among COVID-19 cases. Diffuse alveolar injury, hyaline membrane formation, pneumocyte hyperplasia, microthrombi, fibrin exudation, pulmonary fibrosis, and intra-alveolar hemorrhage are among the most frequently reported pathological findings. DAD has been the most frequently reported among all pulmonary findings with 239 cases (84%). On second thought, these pathological findings should not be seen as one of the COVID-19 attributes without considering other important factors affecting the course of illness like age, symptoms, comorbidities, and management plan. For example, Wichmann et al. investigated the possibility of attributed venous thromboembolism in a cohort of COVID-19 patients. Autopsy results found that deep venous thrombosis was found in 58% of the cases, and this has been linked to COVID-19 while ignoring the fact that the majority of patients suffered from atrial fibrillation, coronary heart disease, and cancers, which have been proven to be decisively determinant factors in developing thromboembolism [31].

On the other hand, similar pathological and autopsy findings have been reported in deceased patients with other coronavirus infections such as MERS-CoV and severe acute respiratory syndrome (SARS). NG et al. published a case report of a deceased 45-year-old patient with MERS-CoV with autopsy findings. Postmortem pulmonary findings included DAD, type 2 pneumocyte hyperplasia, interstitial infiltrate, alveolar fibrin deposits, and prominent hyaline membranes [73]. Alsaad et al. also reported DAD in their report that involved a 33-year-old patient with MERS-CoV [11]. Moreover, Franks et al. reported in their study of eight SARS patients that DAD was the main pathological finding. In contrast, Nicholls et al. reported other findings like the focal deposition of fibrin along the exposed basement membrane [12,74]. On the other hand and in non-coronaviruses pulmonary infections like H1N1 cases, histopathological findings such as septal inflammation, congestion, and thickening of alveolar septae, patchy peripheral hemorrhage, and diffuse alveolar hemorrhage have been reported in different studies [75-78].

Other Organ Findings

Regarding the postmortem cardiac pathology, there were 23 studies with a total number of 87 cases addressing the histopathological findings in the heart. Myocardial hypertrophy, small coronary vessels, cardiac fibrosis, cardiac cell infiltrates, and cardiac amyloidosis were the main findings. Although viral myocarditis has been reported in patients with SARS-CoV-2, lymphocyte infiltrate was found only in one case reported by Buja et al. during immunohistochemical (IHC) staining [29,79]. These pathological findings could be attributed to the comorbidities of affected patients, as most of them suffered from hypertension, diabetes, or coronary heart disease. On the other hand, myocardial edema and fibrosis have been recorded in deceased patients with SARS and MERS-CoV [73,80,81]. While the studies in this review reported that nephrosclerosis, arteriosclerosis, glomerulosclerosis, and acute tubular injury were the most commonly reported findings in the postmortem renal biopsies, other pathological findings like hyaline arteriolosclerosis, patchy interstitial inflammation, and granular casts have been reported in other coronavirus cases like SARS and MERS-CoV [73,82,83]. Regarding the pathological findings in the hepatobiliary system, our review found that hepatic fibrosis, steatosis, cirrhosis, and interstitial inflammations were the main findings. In contrast, other pathological findings were reported in patients with SARS-CoV-1 infection, such as lymphocytic infiltrate and balloon degeneration [84]. As for histopathological findings of the spleen and lymph nodes, lymphocyte depletion and hemophagocytosis of the spleen and lymph nodes were the main findings. Our results were consistent with similar pathological findings from other coronavirus infections [80,85].

Although SARS-CoV-2 has not been detected in the spinal fluid, our study suggests that COVID-19 is capable of infecting the CNS via olfactory and trigeminal nerves, thereby causing cerebral hemorrhage, focal spongiosis, and vascular congestion [86]. On the contrary, in the case of SARS infection, RT-PCR has detected the genomic sequences of the virus in cerebral spinal fluid and brain tissue specimens and was responsible for brain edema and neuronal degeneration [82,87].

Limitations of the study

As is typical of any research, we faced many limitations while conducting the review. Firstly, in this study, we focused on the available studies in certain databases in the first months of the pandemic, and hence government reports and other relevant gray literature were not included in this review; therefore, publication bias is a possibility. Second, due to the scarcity of evidence, we decided to include preprints. These publications had not yet undergone peer review. However, since we assessed the risk of bias in these studies, we feel that the benefits of including the data from these preprints in our review outweigh the risks. Third, we have included only 50 articles, but we cannot ignore the fact that the number of publications is increasing on a daily basis, and we might have missed the recently published ones. Fourth, missing information in some of the published articles has been a challenge. Many articles did not report the basic characteristics of the cases like gender, comorbidities, and clinical course of the disease.

## Conclusions

Postmortem histopathological biopsies play an essential role in helping us understand the pathophysiology of SARS-CoV-2 infection. COVID-19 affects different body organs with different pathological features throughout the course of the infection. Cellular destruction, vascular invasion, and fibrous formation have been identified in the pulmonary, hepatobiliary, and renal systems. Further research is needed to gain a better understanding of the disease and to explore the extent of its effects on different organs and tissues.

## Appendices

Appendix 4: quality checklist of selected studies

https://docs.google.com/spreadsheets/d/1YZZVEUz6uO8Iv9nlZ1OXNCLiux3LUeHbo9ioQFuBsJc/edit?usp=sharing

Appendix 3: microscopic and gross pictures

https://docs.google.com/spreadsheets/d/1d_yuTiLK5P6y18FwEqW57vs2wbEnA5zMz7WfC0dIS2M/edit?usp=sharing

Appendix 2: search strategy

**Table 2:** Appendix 2: search strategy

Database	Search limitation	Concept	Search term/strategy	
			Mesh OR Keywords	
PubMed	Up to August 2020. Adult English search field: title, abstract, and full text	#1	Coronavirus	(“COVID19” [Title/Abstract] OR “COVID-19” [Title/Abstract] OR “coronavirus disease 2019” [Title/Abstract] OR “2019 novel coronavirus infection” [Title/Abstract] OR “coronavirus disease-19” [Title/Abstract] OR “2019-nCoV disease” [Title/Abstract] OR “2019nCoV” [Title/Abstract] OR “2019 novel coronavirus disease” [Title/Abstract] OR “2019-nCoV infection” [Title/Abstract] OR “SARS-CoV-2” [Title/Abstract] OR “SARS2” [Title/Abstract] OR “Wuhan coronavirus" [Title/Abstract]) OR ("COVID-19" [Supplementary Concept] OR "severe acute respiratory syndrome coronavirus 2" [Supplementary Concept])
		#2	Postmortem pathology	"Autopsy"[Mesh] OR "Forensic Pathology"[Mesh] OR “autops*” [Title/Abstract] OR “postmortem” [Title/Abstract] OR “post-mortem” [Title/Abstract] OR “Histopath*” [Title/Abstract] OR “death” [Title/Abstract] OR “specimen” [Title/Abstract] OR “biopsy” [Title/Abstract] OR “cytopathology” [Title/Abstract] OR “immunopathology” [Title/Abstract]
ScienceDirect			(“2019-nCoV” OR “COVID-19” OR “novel coronavirus” OR “2019-nCoV infection” OR “SARS-CoV-2” OR “Wuhan coronavirus") AND (Autopsy OR "immunopathology" OR "autopsies" OR "specimen" OR "histopathology" OR "histopathological" OR "biopsy" OR "Cytopatho")	
Google Scholar			(“2019-nCoV” OR “COVID-19” OR “novel coronavirus” OR “2019-nCoV infection” OR “SARS-CoV-2” OR “Wuhan coronavirus") ("Autopsy" OR "Postmortem" OR pathology OR autopsies OR gravid OR histopathology OR specimen OR cytopathology OR immunopathology)	
medRxiv and bioRxiv			Novel coronavirus AND postmortem pathology	

Appendix 1: PRISMA 2009 checklist used for the review

**Table 3:** Appendix 1: PRISMA 2009 checklist used for the review PRISMA: Preferred Reporting Items for Systematic Reviews and Meta-Analyses

Section/topic	#	Checklist item	Reported on page #
Title	1	Identify the report as a systematic review, meta-analysis, or both	Page 1: (Title)
Abstract			
Structured summary	2	Provide a structured summary including, as applicable: background; objectives; data sources; study eligibility criteria, participants, and interventions; study appraisal and synthesis methods; results; limitations; conclusions and implications of key findings; systematic review registration number	Page 2
Introduction			
Rationale	3	Describe the rationale for the review in the context of what is already known	Page 4 (Introduction)
Objectives	4	Provide an explicit statement of questions being addressed with reference to participants, interventions, comparisons, outcomes, and study design (PICOS)	Page 4 (at end of the Introduction)
Methods			
Protocol and registration	5	Indicate if a review protocol exists, if and where it can be accessed (e.g., web address), and, if available, provide registration information including registration number	A nonpublished protocol available upon request
Eligibility criteria	6	Specify study characteristics (e.g., PICOS, length of follow-up) and report characteristics (e.g., years considered, language, publication status) used as criteria for eligibility, giving rationale	Page 5
Information sources	7	Describe all information sources (e.g., databases with dates of coverage, contact with study authors to identify additional studies) in the search and the date last searched	Page 5
Search	8	Present a full electronic search strategy for at least one database, including any limits used, such that it could be repeated	Appendix 1
Study selection	9	State the process for selecting studies (i.e., screening, eligibility, included in the systematic review, and, if applicable, included in the meta-analysis)	Page 5
Data collection process	10	Describe the method of data extraction from reports (e.g., piloted forms, independently, in duplicate) and any processes for obtaining and confirming data from investigators	Pages 5, 6
Data items	11	List and define all variables for which data were sought (e.g., PICOS, funding sources) and any assumptions and simplifications made	Page 6
Risk of bias in individual studies	12	Describe methods used for assessing the risk of bias of individual studies (including specification of whether this was done at the study or outcome level), and how this information is to be used in any data synthesis	Page 6
Summary measures	13	State the principal summary measures (e.g., risk ratio, difference in means)	Pages 7-11
Synthesis of results	14	Describe the methods of handling data and combining results of studies, if done, including measures of consistency (e.g., I^2^) for each meta-analysis	NA
